# Automated microscopy for malaria diagnosis in a reference laboratory in nonendemic settings

**DOI:** 10.1186/s13071-025-07215-x

**Published:** 2026-01-05

**Authors:** Alexandra Martín-Ramírez, Marta Lanza-Suárez, Pedro Berzosa Díaz, Agustín Benito, Victor Antón-Berenguer, José M. Rubio

**Affiliations:** 1 Malaria and Emerging Parasitic Diseases Laboratory, National Microbiology Centre, Instituto de Salud Carlos III, Madrid, Spain; 2 Centro de Investigación Biomédica en Red de Enfermedades Infecciosas, Instituto de Salud Carlos III, Madrid, Spain; 3 National Centre for Tropical Diseases, Instituto de Salud Carlos III, Madrid, Spain; 4 Department of Microbiology and Parasitology, Hospital Universitario Severo Ochoa, Madrid, Spain

**Keywords:** Malaria, Automated microscopy, Artificial intelligence, Digital microscopy, *Plasmodium*, Diagnosis

## Abstract

**Background:**

Malaria diagnosis plays a key role in case management, control, and elimination strategies. miLab™ is a digital microscopy with a fully integrated, sample-to-result approach, providing automated microscopic analysis of *Plasmodium* parasites and providing parasitemia levels of samples. It uses a deep learning model, a subfield of artificial intelligence (AI) that can differentiate from red blood cells that are infected with the malaria parasite from noninfected cells in blood smears.

The aim of this study is to assess the performance of miLab™ microscopy for malaria diagnosis, in comparison with conventional microscopy and nested-multiplex malaria polymerase chain reaction (NM-PCR), in a malaria reference laboratory in a nonendemic country.

**Methods:**

From 2021 to 2024, 400 samples were analyzed prospectively using automated miLab™ microscopy, with NM-PCR and conventional microscopy as reference methods.

**Results:**

The comparison between the miLab™ device and thin blood smear microscopy showed substantial concordance (90.8%), with a kappa coefficient of 0.8 and sensitivity and specificity values of 92.1% and 89.4%, respectively. The comparison of parasite density showed a significant correlation (correlation coefficient of 0.77), although the parasite counts estimated by the miLab™ device were 11.6% lower than those estimated by conventional microscopy.

The sensitivity and specificity values of the miLab™ platform when compared with those obtained by NM-PCR were 62.8% and 95.4%, respectively; with a concordance value of 68.9% (kappa coefficient 0.4). Of *P. falciparum* infections identified by NM-PCR, 63.4% were accurately identified, and this figure increased to 95.7% if excluding negative results. One *P. vivax*, three *P. ovale*, and one *P. malariae* infections identified by NM-PCR were correctly classified by the miLab™ platform only after expert review of initial “review needed” results.

**Conclusions:**

miLab™ automated microscopy was as sensitive as conventional microscopy but without the need for expert microscopists and with shorter time to results. It is a valuable toolkit for malaria diagnosis in nonendemic settings; however, improvements are required in terms of species identification and parasite quantification.

**Graphical Abstract:**

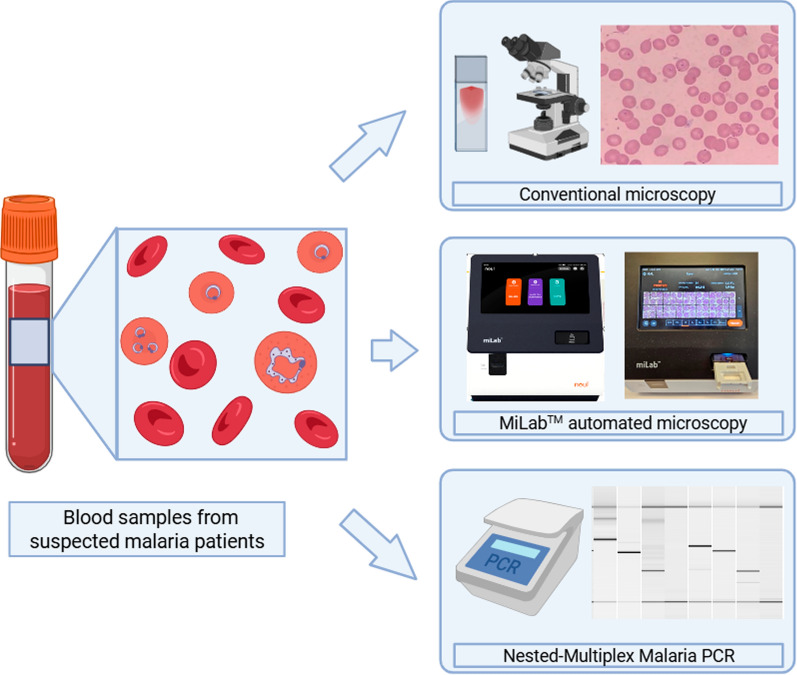

**Supplementary Information:**

The online version contains supplementary material available at 10.1186/s13071-025-07215-x.

## Background

Malaria remains a significant global health burden, with an estimated 263 million cases and 597,000 deaths in 2023 [1]. Accurate diagnosis is critical for case management, disease control, and elimination efforts. Confirmatory testing is mandatory for all suspected cases prior to treatment [2, 3].

Conventional diagnostic methods, including light microscopy and rapid diagnostic tests (RDTs), remain the cornerstone of malaria diagnosis, with thick blood smear microscopy considered the gold standard owing to its low cost and ability to quantify parasitemia [4, 5]. However, microscopy is time-consuming and requires trained personnel. In many endemic areas, quality assurance (QA) programs are lacking or unsustainable, whereas in nonendemic regions, low case numbers lead to a decline in skilled microscopists [6, 7].

These limitations underscore the need for more sensitive and standardized alternatives, such as PCR-based molecular diagnostics [8, 9]. While PCR offers high sensitivity, its implementation in most malaria endemic settings is hindered by high costs, infrastructure requirements, and the need for technical expertise [10].

To overcome these challenges, several innovative diagnostic approaches have been explored. The Global Technical Strategy for Malaria 2016–2030 highlights the importance of evaluating emerging diagnostic technologies to strengthen surveillance and case management [3]. Recent research has focused on improving microscopy using AI-powered digital tools capable of detecting infected red blood cells [11, 12]. Automated systems offer standardized interpretation, faster processing times, and reduced reliance on manual expertise [13].

Smartphone-based solutions integrating AI have shown promise in resource-limited settings, although most remain semi-automated, requiring manual smear preparation and slide handling [14–16]. Fully automated, sample-to-result microscopic systems are rare. One such system is the miLab™ platform, developed by Noul in 2020, which automates blood smear preparation, staining, and scanning of over 200,000 RBCs in 20 min [17]. An integrated AI algorithm identifies *Plasmodium falciparum* and *P. vivax* (including mixed infections of these species), delivering a diagnosis and the estimated parasitemia [18, 19]. The miLab™ platform is designed for various applications, including malaria diagnosis using a modified Romanowsky stain, cytological evaluation with Papanicolaou stain, and blood cell morphology assessment with hematoxylin and eosin stain [17].

While conventional malaria microscopy is effective under optimal conditions, its performance varies widely in the field owing to equipment limitations, inconsistent training, and lack of QA. Nonendemic regions such as Europe also face diagnostic challenges owing to the scarcity of malaria cases and trained experts. Spain has been a malaria-free country since 1964; since then, the majority of cases have been imported, although occasionally some reported cases have no history of travel [20]. In 2022, Spain reported 669 malaria cases, ranking third in the European Union (EU) despite being malaria-free since 1964 [21].

In line with its assigned responsibilities, the Spanish National Reference Laboratory for Malaria (MAPELab) conducted this study to evaluate the diagnostic performance of the miLab™ system in comparison with conventional microscopy and nested multiplex PCR malaria diagnostic methods.

## Methods

### Study design and sample collection

A total of 400 blood samples were prospectively analyzed between 2021 and 2024 using the miLab™ automated microscopy platform (Noul Diagnostics, Republic of Korea). Results were compared against thin blood smear microscopy, using Quick Panoptic staining, and nested multiplex PCR (NM-PCR) for malaria molecular diagnosis [22, 23].

Ethical approval was granted by the Research Ethics Committee of the Instituto de Salud Carlos III, Spain (CEI PI 74_2020). The samples were obtained from travelers or migrant patients who presented with suspected malaria at Spanish hospitals and were subsequently referred to the National Center for Microbiology for diagnostic confirmation.

All blood samples were collected in ethylenediaminetetraacetic acid (EDTA) tubes and transported to the MAPELab within 5 days of collection.

### Automated microscopy using the miLab™ system

Automated microscopy was carried out using the miLab™ diagnostic platform, which employs disposable cartridges comprising three sequential staining zones. The procedure was conducted strictly following the manufacturer’s protocol [4]. In brief, a plastic slide was placed over the cartridge, and 5 µL of whole blood was applied to the designated sample area. The system subsequently performed automated smearing and staining of the entire blood film. After an incubation period of approximately 12–15 min, the device software generated a preliminary diagnostic output. Results were displayed in one of the following categories: positive (subclassified as “*Plasmodium falciparum* positive suspected,” “*P. vivax* positive suspected,” or “Pf/Pv positive suspected”), negative, review needed, or error (Additional file 1: Fig. S1). Upon generation of the result, the operator was given the option to review the diagnostic image and, if required, manually adjust the classification through the user interface.

### Conventional microscopy of malaria

Thin blood smear microscopy was performed on all collected samples using the Quick Panoptic staining method (Química Analítica Aplicada S.A., Spain). Between 2 and 5 µL of blood was used to prepare thin blood smears. Slides were examined by a certified expert microscopist, who was blinded to the results of both miLab™ automated microscopy and PCR assays. Parasite species identification and quantification were conducted assuming a standard concentration of 5,000,000 red blood cells (RBCs) per microliter of blood. A minimum of 100 high-power fields (1000× magnification achieved with a 100× oil immersion objective and a 10× eyepiece) were reviewed prior to categorizing slides as positive or negative.

### Nested-multiplex malaria PCR (NM-PCR)

Genomic DNA for the performance of the nested multiplex PCR (NM-PCR) assay was extracted from whole blood samples using the QIAamp DNA Mini Blood Kit (QIAGEN^®^, Germany) on an automated QIAcube system (QIAGEN®, Germany), following the manufacturer’s protocol. A total of 200 µL of whole blood was processed and eluted in 100 µL of sterile distilled water. The DNA extracts were used immediately for NM-PCR reactions, and the remaining aliquots were stored at 4 °C for subsequent analyses.

NM-PCR was employed as the reference method in this study, as is the reference method commonly used for malaria diagnosis in MAPELab. This method is accredited under the UNE-EN ISO 15189:2022 standard (accreditation no. 175/LE1213) and was performed using the commercial BIOMALAR-2 Gel Form Kit (Biotools B&M Labs, Spain), in accordance with the manufacturer’s instructions and the protocols previously described [22, 23]. The method consists of two sequential multiplex PCR amplifications. The first reaction targets the *Plasmodium* genus and includes a human internal control to assess amplification quality. The second reaction uses the product of the first PCR as a template to detect and differentiate the most common *Plasmodium* species in humans (*P. vivax*, *P. falciparum*, *P. ovale*, *P. malariae*, and *P. knowlesi*).

Amplified products were resolved by capillary electrophoresis using the QIAxcel system (QIAGEN^®^), and species identification was based on the specific fragment sizes corresponding to each *Plasmodium* species.

For the initial PCR reaction, 5 µL of extracted DNA was added to the reaction mix. For the nested PCR, 2 µL of the first-round PCR product was used. The thermocycling conditions for the first PCR included initial denaturation at 94 °C for 7 min, followed by 40 cycles of denaturation at 94 °C for 20 s, annealing at 58 °C for 20 s, and extension at 72 °C for 30 s, with a final extension step at 72 °C for 10 min. The second PCR was performed with an initial denaturation at 94 °C for 5 min, followed by 35 cycles at 94 °C for 15 s, 53 °C for 15 s, and 72 °C for 20 s, concluding with a final extension at 72 °C for 10 min.

### Data analysis

The performance of the miLab™ platform was evaluated in comparison with conventional light microscopy and NM-PCR. Sensitivity, specificity, positive predictive value (PPV), negative predictive value (NPV), and overall diagnostic accuracy were calculated along with their corresponding 95% confidence interval (CI). Method agreement was assessed using Cohen’s kappa statistic (*κ*), interpreted as follows: *κ* ≤ 0, no agreement; 0.01–0.20, slight agreement; 0.21–0.40, fair agreement; 0.41–0.60, moderate agreement; 0.61–0.80, substantial agreement; and 0.81–1.00, almost perfect agreement [24].

To minimize bias in the statistical analysis, a sample was considered positive if the miLab™ device detected *Plasmodium*-infected red blood cells, regardless of species (*P. falciparum* or *P. vivax*). “Review needed” results were reclassified according to the operator’s knowledge into the corresponding category, either a positive or negative result. Results labeled as “error” or “incomplete” were excluded from all statistical analyses. Cases in which miLab™ misidentified the *Plasmodium* species are reported separately.

Comparison of parasite density estimates between miLab™ and thin blood smear microscopy was performed using only samples that tested positive by both methods. The Spearman correlation coefficient (*ρ*) was calculated following verification of data distribution via the Kolmogorov–Smirnov normality test. The strength of correlation was assessed on the basis of the Spearman correlation coefficient value obtained, that is, no correlation (0.0 < 0.1) low correlation (0.1–0.3), moderate correlation (0.3–0.5), high correlation (0.5–0.7), and very high correlation (0.7–1) [25, 26]. Statistical analyses were conducted using SPSS software, version 22.

## Results

The results of *Plasmodium* identification using each diagnostic method are summarized in Table 1. The miLab™ platform yielded 350 valid results (87.5%), while 28 samples (7%) generated errors and 22 samples (5.5%) produced incomplete results (Table 2). Among the valid outcomes provided by miLab™ platform, 156 samples (39%) were identified as positive, comprising 146 *P. falciparum* (Pf), 7 *P. vivax* (Pv), and 3 mixed Pf/Pv infections. A total of 131 samples (32.8%) were classified as negative, and 63 samples (15.8%) were categorized as “review needed” (Table 1).

**Table 1:** Results obtained by the three malaria diagnostic methods tested in this study (miLab™ automated microscopy platform, thin blood smear microscopy, and NM-PCR)

	miLab™ automated microscopy platform^1^	Thin blood smear microscopy	NM-PCR
Positive Pf	146	178	283
Positive Pv	7	6	11
Positive Po	NA	8	14
Positive Pm	NA	5	13
Mixed infection (Pf + Pm)	NA	0	4
Mixed infection (Pf + Pv)	3	0	1
Mixed infection (Pm + Po)	NA	0	1
Mixed infection (Pf + Po)	NA	1	2
Negative	131	192	71
Hemolysis	NA	10	NA
Review needed	63	NA	NA
Error	28	NA	NA
Incomplete	22	NA	NA
Total number of samples	400	400	400

*Pf* *Plasmodium falciparum*, *Pv P. vivax*, *Po* *P. ovale*, *Pm P. malariae*, *NA* not applicable. ^1^miLab™ automated microscopy platform results provided by the system before “review needed” reclassification

**Table 2:** Summary of *Plasmodium* detection results by diagnostic method

Method	Valid results, *n* (%)	Positive, *n* (%)	Negative, *n* (%)	Errors/incomplete/excluded, *n* (%)
miLab™ microscopy	350 (87.5%)	182 (45.5%)	168 (42.0%)	50 (12.5%)
Thin blood smear microscopy	390 (97.5%)	198 (49.5%)	192 (48.0%)	10 (2.5%)
NM-PCR	400 (100%)	329 (82.2%)	71 (17.8%)	0 (0%)

*N* number of samples

The “review needed” samples were further analyzed by an expert microscopist and reclassified as either positive (26 samples; 41.3%) or negative (37 samples; 58.7%). After expert review, the final results from the miLab™ platform were 182 positive samples (45.5%) and 168 negative samples (42%) (Table 2).

Thin blood smear microscopy was successfully performed on 390 samples (97.5%), with 10 samples excluded due to hemolysis (Table 1). These hemolysis samples provided seven “incomplete/error,” one “review needed,” one “negative,” and one *‘P. falciparum* positive suspected” results with the miLab™ platform. Of the valid samples, 198 (49.5%) were identified as positive and 192 (48%) as negative (Table 2). One mixed infection involving *P. falciparum* and *P. ovale* was detected.

Using NM-PCR, all samples were successfully analyzed (Tables 1 and 2). Among them, 329 samples (82.2%) tested positive, while 71 samples (17.8%) were negative. Of the 329 positive cases, 283 were identified as *P. falciparum*, 11 as *P. vivax*, 14 as *P. ovale*, and 13 as *P. malariae* (Table 1). Additionally, eight mixed infections were detected: four *P. falciparum* + *P. malariae*, two *P. falciparum* + *P. ovale*, one *P. malariae* + *P. ovale*, and one *P. falciparum* + *P. vivax*.

The agreement between the miLab™ platform and conventional microscopy was substantial, with an overall concordance of 90.8% and a Cohen’s kappa coefficient of 0.8 (95% CI 0.8–0.9) (Table 3, Additional file 2: Table S1).

**Table 3:** Comparison of the three malaria diagnostic methods

	Sensitivity % (CI 95%)	Specificity % (CI 95%)	PPV % (CI 95%)	NPV % (CI 95%)	Kappa coefficient value (CI 95%)
Comparison of miLab™ and conventional microscopy	92.1% (87.8–96.4%)	89.4% (84.5–94.3%)	90.1% (85.4–94.7%)	91.6% (87.0–96.1%)	0.8 (0.8–0.9)
Comparison of miLab™ and NM-PCR	62.8% (57.0–68.6%)	95.4% (89.5–100.0%)	98.4% (96.2–100.0%)	36.9% (29.3–44.5%)	0.4 (0.3–0.4)
Comparison of conventional microscopy and NM-PCR	62.1% (56.6–67.6%)	100% (99.3–100%)	100% (99.8–100%)	37.0% (29.9–44.1%)	0.4 (0.3–0.5)

*CI* confidence interval

The table shows the agreement between results obtained using the miLab™ automated microscopy platform, thin blood smear microscopy, and NM-PCR, summarizing the sensitivity, specificity, positive predictive value (PPV), negative predictive value (NPV), and the kappa coefficient, with their 95% confidence intervals

The comparison between the miLab™ platform and NM-PCR (Table 4) yielded a sensitivity of 62.8% (95% CI 57.0–68.6%) and a specificity of 95.4% (95% CI 89.5–100.0%). The overall concordance between both methods was 68.9%, with a kappa coefficient of 0.4 (95% CI 0.3–0.4) (Table 3).

**Table 4:** Comparative results between the miLab™ automated microscopy platform and NM-PCR

		NM-PCR								
		Positive Pf	Positive Pv	Positive Po	Positive Pm	Mixed infection (Pf + Pm)	Mixed infection (Pf + Po)	Mixed infection (Pm + Po)	Mixed infection (Pf + Pv)	Negative
miLab™ malaria microscopy^1^	Pf positive suspected	156	0	2	2	1	0	1	0	3
	Pv positive suspected	6	1	0	1	1	0	0	0	0
	Pf/Pv positive suspected	0	0	2	0	0	1	0	0	0
	Po positive	0	0	3	0	0	0	0	0	0
	Pm positive	0	0	0	1	0	0	0	0	0
	*Plasmodium* spp.	1	0	0	0	0	0	0	0	0
	Negative	83	9	4	6	2	1	0	1	62
	Error	22	0	1	0	0	0	0	0	5
	Incomplete	15	1	2	3	0	0	0	0	1

^1^Categorized results after reclassification of “review needed” results by an expert microscopist. *Pf P. falciparum*, *Pv P. vivax*, *Po P. ovale*, *Pm P. malariae*

Conventional thin blood smear microscopy was also compared with NM-PCR (Additional file 2: Table S2), providing 62.1% (95% CI 56.6–67.6%) and 100% (95% CI 99.3–100%) values of sensitivity and specificity, respectively (Table 3).

Regarding miLab™ platform species identification, out of the 246 valid *P. falciparum* results identified by NM-PCR, the miLab™ platform correctly detected 156 cases as *P. falciparum* (Table 4), representing a detection rate of 63.4%. When excluding negative miLab™ results, the identification accuracy increased to 95.7% (156/163). However, six *P. falciparum* infections were misclassified as *P. vivax* by the miLab™ platform (Additional file 3: Fig S2).

One *P. vivax*, three *P. ovale*, and one *P. malariae* infections identified by NM-PCR were correctly classified by the miLab™ platform after expert review of the initial “review needed” result. Additionally, four *P. ovale* infections identified by NM-PCR were misclassified by the miLab™ platform as *P. falciparum* (*n* = 2) or as *P. falciparum*/*P. vivax* mixed infections (*n* = 2). Similarly, three *P. malariae* infections were misclassified as *P. falciparum* (*n* = 2) or *P. vivax* (*n* = 1) (Table 4).

The mixed infection of *P. falciparum* + *P. vivax* detected by NM-PCR was not detected by the miLab™ platform. In contrast, one mixed infection of *P. falciparum* + *P. malariae* was detected as a single *P. falciparum* infection, and one mixed infection of *P. falciparum* + *P. ovale* was detected as mixed *P. falciparum*/*P. vivax* infection. The remaining mixed infections were either misclassified or not detected by the miLab™ platform and reported as negative (Table 4).

**Fig. 1:**
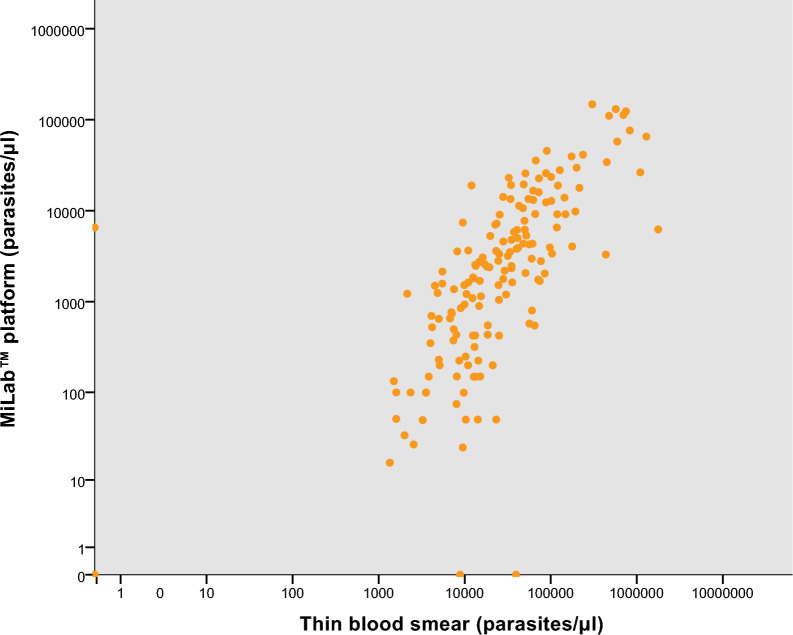
Correlation between parasite density (parasites/µL) measured by the miLab™ platform and expert thin blood smear microscopy for samples positive by both diagnostic methods

Parasitemia values (parasites/µL) obtained using the miLab™ platform were compared with those determined by thin blood smear microscopy in 161 samples. A significant Spearman correlation was observed (*p* = 0.0), with a correlation coefficient of 0.77 (Fig. 1), indicating a very high positive correlation. On average, parasite counts estimated by the miLab™ device were 11.6% lower than those obtained by conventional microscopy.

## Discussion

Timely diagnosis of malaria is critical for appropriate patient treatment and management. In this study, the performance of the miLab™ platform for malaria diagnosis was prospectively evaluated at the Spanish National Malaria Reference Laboratory, located in a nonendemic malaria area. Its diagnostic accuracy was compared with two established methods: conventional light microscopy using thin blood smears, as a conventional method for malaria diagnosis, and nested multiplex malaria PCR (NM-PCR), as a molecular method with increased sensitivity compared with conventional methods and able to detect submicroscopic malaria infections.

The comparison of miLab™ digital microscope with conventional microscopy yielded high values of sensitivity and specificity. Even 15 out of 18 samples that were classified as positive by miLab™ microscopy (6 after expert microscopist reclassification) but negative by conventional microscopy were positive by NM-PCR (12 *P. falciparum*, 1 *P. vivax*, 1 *P. ovale*, and 1 *Plasmodium* spp.), pointing out the proper diagnosis made by miLab™ and incorrect diagnosis by thin blood smear microscopy in those samples. The similar performance of the miLab™ device to blood smear microscopy found in this study was also found in previous articles [4, 18], although a previous study showed increased sensitivity of miLab™ microscopy compared with conventional microscopy at a health post in Ethiopia and with expert microscopy in Ghana [27].

The comparison of the miLab™ platform and NM-PCR results showed moderate values of sensitivity, while the specificity was high. However, the kappa coefficient showed fair agreement. Similar results of sensitivity, specificity, and kappa coefficient were obtained for thin blood smear microscopy in comparison with NM-PCR. These results can be explained by the higher sensitivity of molecular methods [8, 28], and the characteristics of the setting where the study was performed. The MAPELab acts as the malaria reference laboratory where samples from patients suspected of having malaria are sent from different Spanish hospitals. These samples mainly include travelers and visiting friends and relatives patients (VFRs), most of them with suspected malaria symptoms but others without clinical symptoms returning from endemic areas. In addition, malaria symptoms are very unspecific and may overlap with other infectious diseases [29–31], leading to the detection of submicroscopic malaria infections when molecular techniques are used in a reference center, even in cases where other pathogens are primarily responsible for the clinical symptoms of the patients. Submicroscopic malaria can be defined as patients with negative blood smears and detection of *Plasmodium* spp. DNA by a molecular method [32]. A previous study showed a prevalence of submicroscopic malaria in afebrile immigrants in Spain of 5.7% [32]. Furthermore, in malaria-endemic areas, submicroscopic malaria accounts for a high percentage of infections, with studies showing detection rates of 14.2% in Kenya [33], 1.2% and 12.8% in different areas in Ethiopia [34], and 11.8% in Cameroon [35]. In fact, 90 out of 106 samples that were negative by the miLab™ platform but positive by NM-PCR were also negative by conventional microscopy, suggesting that, irrespective of clinical symptoms, these could be considered submicroscopic malaria infections. Then, if these negative samples with low parasitemias are not taken into account, the miLab™ automated microscopy sensitivity would increase to 91.8%.

Among samples positive by the miLab™ device, it assigned the wrong species to 13 of 175 monoinfections detected by NM-PCR, and in 1 sample the species could not be determined by the operator after a “review needed” result. Accuracy in the identification of *P. falciparum* infections was high, with 95.7% of these samples providing a positive result, and just six samples classified such as *P. vivax* infections and another one as *Plasmodium* spp. The main reason for this *P. vivax* misidentification was the detection of gametocyte forms, which the device classified as *P. vivax*. Nevertheless, this misidentification occurred in samples assessed during 2021, with the first version of the device, but not with successive versions. The *Plasmodium* spp. sample was a “review needed” infection where the expert microscopist could not achieve species identification owing to the low number of parasite forms. The identification of *P. vivax*, *P. ovale*, *P. malariae*, and mixed malaria infections was not successful with the miLab™ device; however, operator review of “review needed” results could achieve the identification of some of these species. Among the 11 *P. vivax* infections detected by NM-PCR, miLab™ provided 9 negative results, 1 Pv positive suspected (after reclassification of a “review needed” result), and 1 incomplete result. Conventional microscopy did identify six *P. vivax* infections (four negatives, the Pv positive suspected result, and one incomplete result by miLab™), while the other five infections were negative. The misidentification of *P. ovale* and *P. malariae* parasites responds to the design of the miLab™ algorithm, which has not been trained to identify them with the current AI model, so these are not yet included as detectable targets. Nevertheless, operator review of “review needed” results provided the identification of three *P. ovale* and one *P. malariae* infections. This is also the reason for the low accuracy in detecting mixed malaria infections. The miLab™ platform provided three “Pf/Pv suspected” results, one corresponding to a sample positive for *P. falciparum* and *P. ovale* by NM-PCR, correctly identified by conventional microscopy, and two corresponding to *P. ovale* infections, detected by conventional microscopy, too. The other mixed infections detected by NM-PCR were not detected by either automated or conventional microscopy. The higher sensitivity of molecular methods in the identification of mixed infections compared with microscopy is well known [36]. In addition, just one of the mixed infections in this study comprised *P. falciparum* and *P. vivax* parasites, the two species which are the target of the miLab™ device, and this sample provided a negative result by both microscopy techniques, suggesting a low level of parasites. The accuracy of the miLab™ device in detecting mixed infections in this study was low, although in a previous study, mixed infections identification by the miLab™ platform was moderate, with 5 of 18 mixed-species infections correctly identified [27].

In addition to positive (*P. falciparum* positive suspected, *P. vivax* positive suspected, and Pf/Pv positive suspected) and negative results, the miLab™ device provides “incomplete,” “error,” and “review needed” results. The number of incomplete and error results found in this study was quite similar (22 and 28 samples, respectively). The occurrence of these results may be related to the delay in receiving the samples at the reference laboratory from the hospitals, which may lead to blood hemolysis or to failures in storage and transportation of the samples. “Review needed” results, given for 15.8% of samples, were more frequent in samples positive by NM-PCR (38/63). This classification of the sample made by the miLab™ device allows the operator to review the suspected results and categorize them as either negative or positive, which has been shown to increase the specificity of the automated microscope [4]. In this study, such operator review reclassified 25 samples negative by NM-PCR as negative results, while the remaining 38 “review needed” samples were classified as either positive (26 samples) or negative (12 samples).

Parasite density calculations obtained by both microscopy techniques, viz. automated and conventional, were compared, showing a high correlation coefficient. However, the calculated parasitemia was relatively lower with the miLab™ device than with expert conventional microscopy. One of the possible reasons for this difference is the lack of identification of parasite shapes other than rings. This may also be influenced by the delay in receiving the samples at a reference center, about 2–5 days from blood collection at Spanish hospitals. This might alter parasite forms [37], leading to failure to identify or misidentification of *Plasmodium* parasites, which may underestimate parasitemia values.

Some limitations of this study may have influenced the results. The main one is the delay in receiving some samples. This delay might alter parasite forms and affect the ability of the miLab™ device to provide results. However, the operator is able to verify the result provided by the automated microscopy when it is displayed on the screen and change it if necessary. Also, improvements in the miLab™ diagnostic algorithm are ongoing, which will provide improvements in parasite species identification [27]. In addition, the low number of *P. falciparum* + *P. vivax* mixed infections (just one sample) limited the assessment of mixed infections identification by the miLab™ platform. This low number of *P. falciparum* + *P. vivax* mixed infections derives from the fact that the majority of the migrant population in Spain comes from sub-Saharan Africa, where *P. vivax* and therefore mixed *P. vivax* infections are less common [38].

The operational characteristics of the miLab™ platform make it a suitable tool for use in both endemic and nonendemic malaria settings. In nonendemic areas, the typically low demand for malaria diagnostics often leads to a decline in the number of expert microscopists. Conversely, many endemic countries face increasing case numbers but lack the infrastructure and resources to systematically train and retain skilled personnel, making it difficult to ensure consistent microscopy quality.

## Conclusions

In this study, the miLab™ platform demonstrated sensitivity comparable to that of conventional microscopy, and it may serve as a valuable tool for malaria diagnosis in both endemic and nonendemic malaria settings. Furthermore, performing the test shortly after sample collection could address some of the limitations observed in this study related to delayed sample processing, which affected parasite detection.

Overall, the miLab™ platform offers a promising solution for malaria diagnosis without the need for expert microscopists. However, further improvements are needed in terms of species identification and parasite quantification to enhance its diagnostic utility.

## Supplementary Information

Supplementary material 1. The miLab™ device screen showing a *Pf* positive suspected result (**A**) and a negative result (**B**) after automated analysis

Supplementary material 2. Additional file 2: Table S1. Comparative results between the miLab™ automated microscopy platform and the thin blood smear microscopy (n=400). Table S2. Comparative results between the thin blood smear microscopy and the NM-PCR (*n* = 400)

Supplementary material 3. Additional file 3: Fig S2: The figure shows two *P. falciparum* samples misidentified as *P. vivax* by the miLab™ platform (one at the top and one at the bottom). **A** Results from the miLab™ device showing infected red blood cells with ring forms and the identification provided by the automated microscope: “*P. vivax* suspected”. **B**: *P. vivax* gametocyte detected by the miLab™ device in the sample analyzed in A. **C** miLab™ device screen showing infected erythrocytes with ring forms and the “*P. vivax* suspected” result provided by the miLab™ microscope. **D** *P. vivax* gametocyte detected by the miLab™ platform in the sample shown in C

## Data Availability

Data supporting the main conclusions of this study are included in the manuscript and its Supplementary Information.
