# Supplementary figures and images for: Automated microscopy for malaria diagnosis in a reference laboratory in nonendemic settings
Source: Parasit Vectors. 2026 Jan 5;19:67. doi: 10.1186/s13071-025-07215-x (PMC12870222; doi:10.1186/s13071-025-07215-x)

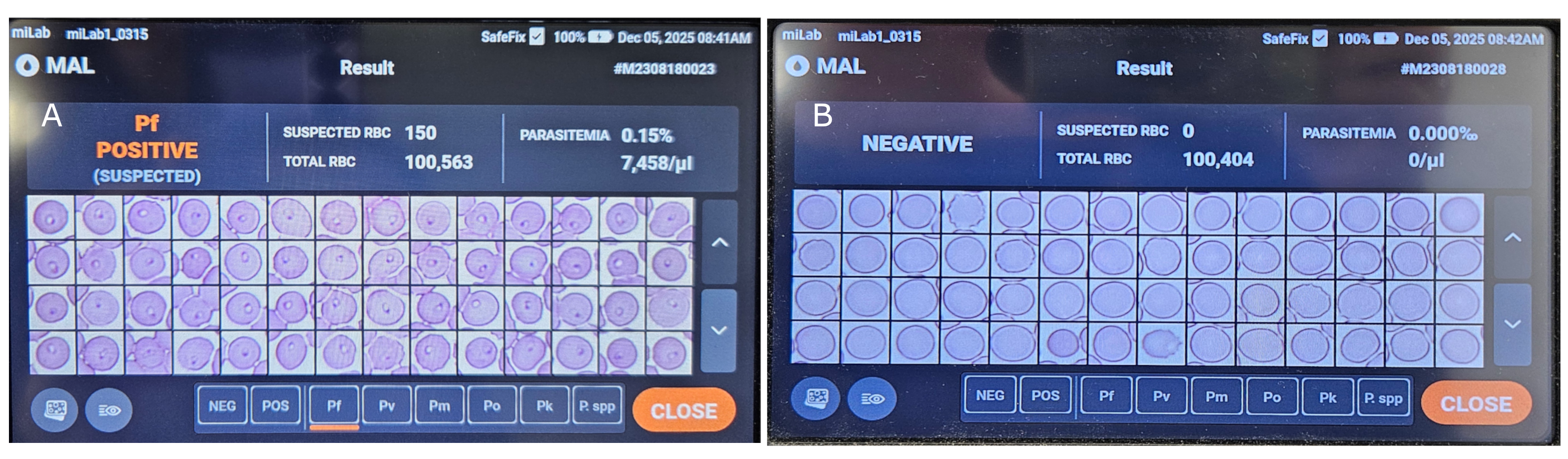

Supplement: Supplementary file 1 — Supplementary material 1. The miLab™ device screen showing a Pf positive suspected result (A) and a negative result (B) after automated analysis [file 13071_2025_7215_MOESM1_ESM.png]

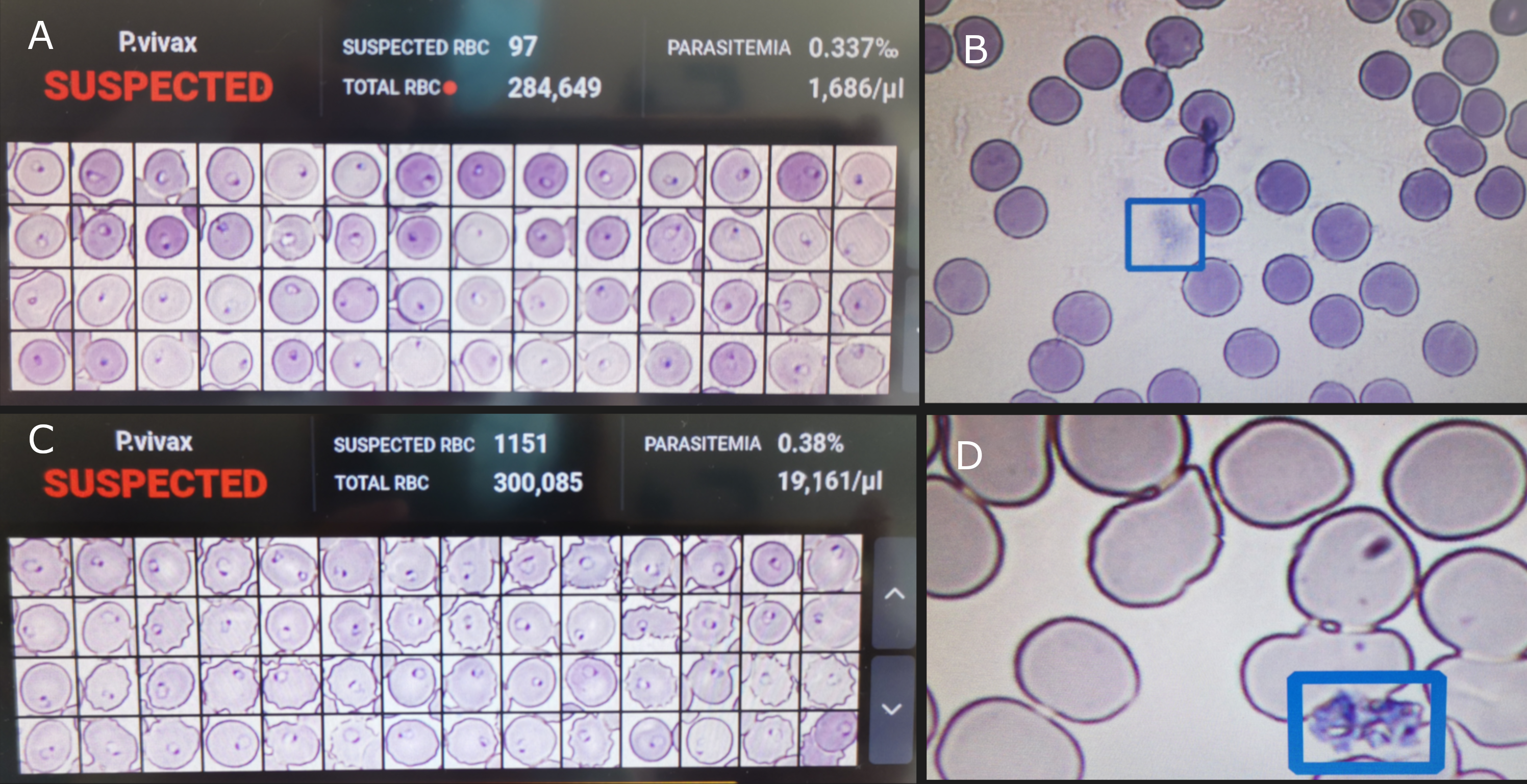

Supplement: Supplementary file 3 — Supplementary material 3. Additional file 3: Fig S2: The figure shows two P. falciparum samples misidentified as P. vivax by the miLab™ platform (one at the top and one at the bottom). A Results from the miLab™ device showing infected red blood cells with ring forms and the identification provided by the automated microscope: “P. vivax suspected”. B: P. vivax gametocyte detected by the miLab™ device in the sample analyzed in A. C miLab™ device screen showing infected erythrocytes with ring forms and the “P. vivax suspected” result provided by the miLab™ microscope. D P. vivax gametocyte detected by the miLab™ platform in the sample shown in C [file 13071_2025_7215_MOESM3_ESM.png]
