# Supplementary material for: Automated microscopy for malaria diagnosis in a reference laboratory in nonendemic settings
Source: Parasit Vectors. 2026 Jan 5;19:67. doi: 10.1186/s13071-025-07215-x (PMC12870222; doi:10.1186/s13071-025-07215-x)
Supplement: Supplementary file 2 — Supplementary material 2. Additional file 2: Table S1. Comparative results between the miLab™ automated microscopy platform and the thin blood smear microscopy (n=400). Table S2. Comparative results between the thin blood smear microscopy and the NM-PCR (n = 400) [file 13071_2025_7215_MOESM2_ESM.docx]

**Supplementary Information**

**Additional file 2: Table S1**. Comparative results between the MiLab™ automated microscopy platform and the thin blood smear microscopy (n=400). **Table S2.** Comparative results between the thin blood smear microscopy and the NM-PCR (n=400).

Additional file 2: Table S1.

|  |  | **Thin blood smear microscopy** | | | | | | |
| --- | --- | --- | --- | --- | --- | --- | --- | --- |
|  |  | **Positive Pf** | **Positive Pv** | **Positive Po** | **Positive Pm** | **Positive Pf+Po** | **Negative** | **Hemolysis** |
| **miLab^TM^ platform^1^** | **Pf positive suspected** | 144 | 0 | 3 | 2 | 0 | 15 | 1 |
|  | **Pv positive suspected** | 6 | 1 | 0 | 1 | 0 | 1 | 0 |
|  | **Pf/Pv positive suspected** | 0 | 0 | 2 | 0 | 1 | 0 | 0 |
|  | **Po positive** | 0 | 0 | 2 | 0 | 0 | 1 | 0 |
|  | **Pm positive** | 0 | 0 | 0 | 1 | 0 | 0 | 0 |
|  | ***Plasmodium* spp.** | 0 | 0 | 0 | 0 | 0 | 1 | 0 |
|  | **Negative** | 8 | 4 | 1 | 1 | 0 | 152 | 2 |
|  | **Error** | 12 | 0 | 0 | 0 | 0 | 13 | 3 |
|  | **Incomplete** | 8 | 1 | 0 | 0 | 0 | 9 | 4 |
|  | **Total samples**  **(n=400)** | 178 | 6 | 8 | 5 | 1 | 192 | 10 |

^1^Categorised results after reclassification of ‘review needed’ results by an expert microscopist. Pf: *P. falciparum,* Pv*: P. vivax,* Po*: P. ovale,* Pm*: P. malariae.*

Additional file 2: Table S2.

|  |  | **NM-PCR** | | | | | | | | |
| --- | --- | --- | --- | --- | --- | --- | --- | --- | --- | --- |
|  |  | **Positive  Pf** | **Positive  Pv** | **Positive  Po** | **Positive  Pm** | **Mixed infection  (Pf+Pm)** | **Mixed infection  (Pf+Po)** | **Mixed infection  (Pm+Po)** | **Mixed infection  (Pf+Pv)** | **Negative** |
| **Thin blood smear microscopy** | **Positive Pf** | 177 | 0 | 0 | 0 | 1 | 0 | 0 | 0 | 0 |
|  | **Positive Pv** | 0 | 6 | 0 | 0 | 0 | 0 | 0 | 0 | 0 |
|  | **Positive Po** | 0 | 0 | 7 | 0 | 0 | 0 | 1 | 0 | 0 |
|  | **Positive Pm** | 0 | 0 | 0 | 3 | 2 | 0 | 0 | 0 | 0 |
|  | **Mixta Pf+Po** | 0 | 0 | 0 | 0 | 0 | 1 | 0 | 0 | 0 |
|  | **Negative** | 98 | 5 | 6 | 9 | 1 | 1 | 0 | 1 | 71 |
|  | **Hemolysis** | 8 | 0 | 1 | 1 | 0 | 0 | 0 | 0 | 0 |
|  | **Total samples**  **(n=400)** | 283 | 11 | 14 | 13 | 4 | 2 | 1 | 1 | 71 |

Pf: *P. falciparum,* Pv*: P. vivax,* Po*: P. ovale,* Pm*: P. malariae.*
